# Supplementary material for: Allosteric regulation and crystallographic fragment screening of SARS-CoV-2 NSP15 endoribonuclease
Source: Nucleic Acids Res. 2023 Apr 28;51(10):5255–70. doi: 10.1093/nar/gkad314 (PMC10250223; doi:10.1093/nar/gkad314)
Supplement: gkad314_Supplemental_Files [file gkad314_supplemental_files.zip › Supplementary datasets.zip!/134/Refine_9/01-BUSTER/Cycle-4/shell.01/R_Rfree.html]

# Rxpct factors in resolution bins at cycle 0


|  |  |  |  |  |  |  |  |  |
| --- | --- | --- | --- | --- | --- | --- | --- | --- |
|  | **<d> (Å)**  Rxpct\_Work | Rxpct\_Free | Rxpct\_All | |  |  |  |  |
|  | 15.4464 | 0.3419 | 0.3884 | 0.3437 |  |
|  | 8.9180 | 0.2237 | 0.2365 | 0.2244 |  |
|  | 6.9078 | 0.2251 | 0.2646 | 0.2273 |  |
|  | 5.8382 | 0.2211 | 0.2843 | 0.2232 |  |
|  | 5.1488 | 0.1765 | 0.2049 | 0.1780 |  |
|  | 4.6573 | 0.1564 | 0.1887 | 0.1583 |  |
|  | 4.2841 | 0.1628 | 0.1855 | 0.1640 |  |
|  | 3.9882 | 0.1618 | 0.2134 | 0.1642 |  |
|  | 3.7463 | 0.1675 | 0.2244 | 0.1702 |  |
|  | 3.5436 | 0.1691 | 0.2027 | 0.1709 |  |
|  | 3.3707 | 0.1749 | 0.2182 | 0.1773 |  |
|  | 3.2208 | 0.1818 | 0.2168 | 0.1837 |  |
|  | 3.0893 | 0.1910 | 0.2277 | 0.1927 |  |
|  | 2.9727 | 0.1924 | 0.2022 | 0.1927 |  |
|  | 2.8683 | 0.1970 | 0.1986 | 0.1971 |  |
|  | 2.7743 | 0.1968 | 0.2382 | 0.1987 |  |
|  | 2.6889 | 0.1938 | 0.1992 | 0.1941 |  |
|  | 2.6109 | 0.2022 | 0.2250 | 0.2035 |  |
|  | 2.5394 | 0.2144 | 0.2308 | 0.2154 |  |
|  | 2.4734 | 0.2282 | 0.2452 | 0.2292 |  |
|  | **Overall** | 0.1918 | 0.2225 | 0.1934 |  |

# Rxpct factors in resolution bins at cycle 1


|  |  |  |  |  |  |  |  |  |
| --- | --- | --- | --- | --- | --- | --- | --- | --- |
|  | **<d> (Å)**  Rxpct\_Work | Rxpct\_Free | Rxpct\_All | |  |  |  |  |
|  | 15.4464 | 0.3420 | 0.3883 | 0.3437 |  |
|  | 8.9180 | 0.2237 | 0.2364 | 0.2245 |  |
|  | 6.9078 | 0.2251 | 0.2644 | 0.2273 |  |
|  | 5.8382 | 0.2210 | 0.2849 | 0.2232 |  |
|  | 5.1488 | 0.1763 | 0.2046 | 0.1778 |  |
|  | 4.6573 | 0.1563 | 0.1886 | 0.1581 |  |
|  | 4.2841 | 0.1625 | 0.1851 | 0.1637 |  |
|  | 3.9882 | 0.1615 | 0.2134 | 0.1639 |  |
|  | 3.7463 | 0.1672 | 0.2250 | 0.1699 |  |
|  | 3.5436 | 0.1689 | 0.2027 | 0.1707 |  |
|  | 3.3707 | 0.1746 | 0.2177 | 0.1770 |  |
|  | 3.2208 | 0.1818 | 0.2165 | 0.1836 |  |
|  | 3.0893 | 0.1909 | 0.2278 | 0.1926 |  |
|  | 2.9727 | 0.1923 | 0.2025 | 0.1927 |  |
|  | 2.8683 | 0.1969 | 0.1989 | 0.1970 |  |
|  | 2.7743 | 0.1967 | 0.2386 | 0.1986 |  |
|  | 2.6889 | 0.1937 | 0.1992 | 0.1940 |  |
|  | 2.6109 | 0.2021 | 0.2247 | 0.2034 |  |
|  | 2.5394 | 0.2140 | 0.2306 | 0.2150 |  |
|  | 2.4734 | 0.2279 | 0.2452 | 0.2289 |  |
|  | **Overall** | 0.1916 | 0.2225 | 0.1932 |  |

# Rxpct factors in resolution bins at cycle 2


|  |  |  |  |  |  |  |  |  |
| --- | --- | --- | --- | --- | --- | --- | --- | --- |
|  | **<d> (Å)**  Rxpct\_Work | Rxpct\_Free | Rxpct\_All | |  |  |  |  |
|  | 15.4464 | 0.3420 | 0.3882 | 0.3438 |  |
|  | 8.9180 | 0.2237 | 0.2364 | 0.2245 |  |
|  | 6.9078 | 0.2250 | 0.2642 | 0.2272 |  |
|  | 5.8382 | 0.2209 | 0.2853 | 0.2231 |  |
|  | 5.1488 | 0.1761 | 0.2044 | 0.1776 |  |
|  | 4.6573 | 0.1561 | 0.1886 | 0.1580 |  |
|  | 4.2841 | 0.1623 | 0.1848 | 0.1635 |  |
|  | 3.9882 | 0.1614 | 0.2135 | 0.1638 |  |
|  | 3.7463 | 0.1671 | 0.2253 | 0.1698 |  |
|  | 3.5436 | 0.1688 | 0.2029 | 0.1707 |  |
|  | 3.3707 | 0.1745 | 0.2175 | 0.1769 |  |
|  | 3.2208 | 0.1818 | 0.2163 | 0.1836 |  |
|  | 3.0893 | 0.1909 | 0.2279 | 0.1926 |  |
|  | 2.9727 | 0.1923 | 0.2027 | 0.1928 |  |
|  | 2.8683 | 0.1970 | 0.1990 | 0.1971 |  |
|  | 2.7743 | 0.1967 | 0.2389 | 0.1986 |  |
|  | 2.6889 | 0.1937 | 0.1995 | 0.1940 |  |
|  | 2.6109 | 0.2021 | 0.2247 | 0.2034 |  |
|  | 2.5394 | 0.2140 | 0.2306 | 0.2150 |  |
|  | 2.4734 | 0.2279 | 0.2451 | 0.2288 |  |
|  | **Overall** | 0.1916 | 0.2225 | 0.1931 |  |

# Rxpct factors in resolution bins at cycle 3


|  |  |  |  |  |  |  |  |  |
| --- | --- | --- | --- | --- | --- | --- | --- | --- |
|  | **<d> (Å)**  Rxpct\_Work | Rxpct\_Free | Rxpct\_All | |  |  |  |  |
|  | 15.4464 | 0.3337 | 0.3890 | 0.3358 |  |
|  | 8.9180 | 0.2201 | 0.2415 | 0.2214 |  |
|  | 6.9078 | 0.2164 | 0.2584 | 0.2188 |  |
|  | 5.8382 | 0.2135 | 0.2929 | 0.2162 |  |
|  | 5.1488 | 0.1702 | 0.2087 | 0.1723 |  |
|  | 4.6573 | 0.1532 | 0.1853 | 0.1550 |  |
|  | 4.2841 | 0.1586 | 0.1836 | 0.1600 |  |
|  | 3.9882 | 0.1569 | 0.2195 | 0.1598 |  |
|  | 3.7463 | 0.1623 | 0.2322 | 0.1656 |  |
|  | 3.5436 | 0.1672 | 0.2042 | 0.1692 |  |
|  | 3.3707 | 0.1727 | 0.2100 | 0.1748 |  |
|  | 3.2208 | 0.1821 | 0.2157 | 0.1839 |  |
|  | 3.0893 | 0.1919 | 0.2297 | 0.1937 |  |
|  | 2.9727 | 0.1938 | 0.2003 | 0.1941 |  |
|  | 2.8683 | 0.1983 | 0.2076 | 0.1988 |  |
|  | 2.7743 | 0.1960 | 0.2497 | 0.1985 |  |
|  | 2.6889 | 0.1942 | 0.2049 | 0.1948 |  |
|  | 2.6109 | 0.2037 | 0.2242 | 0.2049 |  |
|  | 2.5394 | 0.2145 | 0.2354 | 0.2159 |  |
|  | 2.4734 | 0.2304 | 0.2486 | 0.2314 |  |
|  | **Overall** | 0.1885 | 0.2241 | 0.1903 |  |
